# A Patient's Sanatorium Experiences

**Published:** 1913-09-06

**Authors:** 


*To the Editor of* The Hospital.

### A Patient's Sanatorium Experiences.

*To the Editor of* The Hospital.

Sir,—I have read with interest the two experiences of patients in to-day's Hospital, I should like to tell you my experience. I am a trained certificated nurse. At the age of thirty-nine I broke down with tuberculosis, and being unable to pay the fees charged in most sanatoriums ten years ago, I was admitted into the wards of a London chest hospital for a period of thirteen weeks. I received exactly the same treatment as every other patient, and I shall ever be grateful for the many kindnesses received. I had pleurisy and effusion, and was most carefully and kindly nursed. After leaving there I went to a sanatorium at the sea for the same period, and here again I received the same kind care and attention. The food in each was good, and more varied than I should have expected. In each of these places there was a certain amount of grumbling, often at the food, but I think when people have nothing to do every little thing is magnified. Should I ever break down again I would gladly go back to either.

Some years after I had some eye trouble, and as we were in the country it was deemed advisable to send me to the ophthalmic wards of a London hospital. Here, to my great surprise, I was treated as if I were a member of their own staff. Sister apologised for not being able to give me a special room. (I did not expect that.) I was given a corner bed with a screen at the side. My meals were specially served, being sent from the paying part of the building. When I was allowed up I was allowed to sometimes go to the nurses' home, and was often asked into sisters' room, privileges that I was careful not to abuse, and my stay there was an exceedingly happy one. I shall never forget the extreme kindness and the skilful nursing. Although I have been unfortunate as regards my health, I seem to have been most fortunate in respect to the hospitals in which I received treatment.—Yours faithfully,

Matron.

August 30.

